# Design of Nonlinear Autoregressive Exogenous Model Based Intelligence Computing for Efficient State Estimation of Underwater Passive Target

**DOI:** 10.3390/e23050550

**Published:** 2021-04-29

**Authors:** Wasiq Ali, Wasim Ullah Khan, Muhammad Asif Zahoor Raja, Yigang He, Yaan Li

**Affiliations:** 1 School of Marine Science and Technology, Northwestern Polytechnical University, Xi’an 710072, China; wasiqali@mail.nwpu.edu.cn (W.A.); liyaan@nwpu.edu.cn (Y.L.); 2 Department of Electrical and Computer Engineering, COMSATS University Islamabad, Attock Campus, Attock 43600, Pakistan; 3 School of Electrical Engineering and Automation, Wuhan University, Wuhan 430072, China; 4 Future Technology Research Center, National Yunlin University of Science and Technology, 123 University Road, Section 3, Douliou, Yunlin 64002, Taiwan; rajamaz@yuntech.edu.tw

**Keywords:** nonlinear autoregressive with exogenous input (NARX), state estimation, artificial neural network, measurement noise, nonlinear filtering, intelligent computing

## Abstract

In this study, an intelligent computing paradigm built on a nonlinear autoregressive exogenous (NARX) feedback neural network model with the strength of deep learning is presented for accurate state estimation of an underwater passive target. In underwater scenarios, real-time motion parameters of passive objects are usually extracted with nonlinear filtering techniques. In filtering algorithms, nonlinear passive measurements are associated with linear kinetics of the target, governing by state space methodology. To improve tracking accuracy, effective feature estimation and minimizing position error of dynamic passive objects, the strength of NARX based supervised learning is exploited. Dynamic artificial neural networks, which contain tapped delay lines, are suitable for predicting the future state of the underwater passive object. Neural networks-based intelligence computing is effectively applied for estimating the real-time actual state of a passive moving object, which follows a semi-curved path. Performance analysis of NARX based neural networks is evaluated for six different scenarios of standard deviation of white Gaussian measurement noise by following bearings only tracking phenomena. Root mean square error between estimated and real position of the passive target in rectangular coordinates is computed for evaluating the worth of the proposed NARX feedback neural network scheme. The Monte Carlo simulations are conducted and the results certify the capability of the intelligence computing over conventional nonlinear filtering algorithms such as spherical radial cubature Kalman filter and unscented Kalman filter for given state estimation model.

## 1. Introduction

The basic aim in state estimation of the underwater passive target is to accurately approximate the motion variables and target’s actual trajectory by obtaining valuable data from noisy measurements of hydrophones [1,2]. Real-time state estimation has many practical applications in both civil and military fields like target detection [3], aircraft surveillance [4], navigation [5], precise guidance [6] and object localization [7]. To achieve accurate state estimation in these real-life problems, nonlinear filtering algorithms are widely applied by the research community in the last two decades because of their efficiency and robustness [8]. Consequently, the accuracy of state estimation phenomena is largely dependent on the convergence of a specific filtering algorithm [9]. Usually, Bayesian filtering techniques offer better results in state prediction problems, where real-time parameters like velocity, position and possibly trajectory of the dynamic target are extracted [10]. In nonlinear systems, Bayesian filtering algorithms are applied in a recursive manner to achieve an efficient estimation of state parameters [11]. Bearings-only passive state approximation problems in complex ocean environment are specifically solved with nonlinear filtering techniques and this scheme is usually referred to target motion analysis [12].

In the literature, the famous Kalman filter (KF), following Bayesian methodology, is widely applied to solve state estimation problems for linear systems [13]. In this filter both passive bearings and estimated dynamics of a moving vehicle are used to compute the next probability density function (PDF) of object state. In KF, optimum state estimation of the dynamic target is calculated using the root mean square error (RMSE) criterion [14]. Practically, in a complex underwater atmosphere, the performance of KF declines due to nonlinear and undetermined characteristics of the observation model [15]. Therefore, this phenomenon reduces the applications of KF in practical nonlinear systems. To solve the issue of non linearity in the measurement model, researchers proposed a Taylor series based first partial optimal nonlinear variant of KF known as extended Kalman filter (EKF) [16]. Still, time-varying and undefined noise covariance in real-time state estimation problems diverge the performance of EKF. To obtain precise position estimation of a passive object, many adaptive EKF schemes have been established by the research community that have the ability to update weights of noise covariance throughout the prediction process [17]. Although better covariance approximation offers an adaptive EKF algorithm, still many robust nonlinear systems produce poor posterior PDF of state vector by Taylor series approximation.

A serial Monte Carlo simulations-based technique, known as particle filter (PF), was introduced to handle the issue of non linearity by applying the bulk of particles for evaluating posterior density distribution [18]. PF shows a satisfactory performance in nonlinear location estimation problems but at the cost of complexity and high numerical computations. As an alternative to linearization, the unscented transform method is used in unscented Kalman filter (UKF) to solve state error covariance by propagating sigma points [19]. UKF algorithm has the advantage of less computational cost than PF and high filtering precision than EKF in nonlinear systems [20]. Along with these filtering techniques, researchers also proposed many nonlinear filters like cubature Kalman filter [21], Gaussian filters [22], Gauss-Hermite quadrature filter [23], the sparse grid quadrature filter [24], shifted Rayleigh filter [25] and ensemble Kalman filter [26]. Some scholars integrate information entropy theory with parameter extraction techniques to investigate characteristics of underwater object. Also in multi target tracking the maximum entropy theory has been combined with JPDA method [27]. Hence, information entropy theory has also important research value in underwater passive target tracking.

In the last decade, researchers have proposed many soft computing methodologies by following artificial intelligence studies. In these intelligence computing schemes, the strength of artificial neural networks (ANN) and optimization with local and global search algorithms are exploited [28]. The spectrum of ANN modeling is spreading extensively in various real-life studies such as electromagnetism [29], nonlinear optics [30], nanotechnology [31], bioinformatics [32], mathematical equations [33], meteorology [34], fluid dynamics [35], thermodynamics [36], rotating electrical machine [37], electric motors [38], atomic physics [39], plasma physics [40] and astrophysics [41]. ANNs show outstanding and significant performance in real-life applications and emerged as one of the dominating models [42]. In the comparison with other methods, ANNs have provided a better convergence rate as described in several relevant studies [39,40,41]. ANNs have the potential to produce excellent results in non-linear data modeling.

In many practical engineering systems, whose characteristics are time-varying and which rely on their present state, non linearity is a big challenge to handle. In these systems, the NARX neural network paradigm can be convenient [43,44]. Recently, NARX has found wide use in a variety of real life applications such as quantitative investment forecasting [45], air quality forecasting [46], predicting marine engine performance [47], intelligent proportional integral derivative systems [48] and prediction of solar radiation [49]. The major benefit of this soft computing technique is the potential to handle dynamic inputs expressed by time-series data sets. Time series prediction by NARX is a variable approach, in which information about the procedure that initializes the time series is not important. NARX can be applied for the prediction of the next instant for any input signal. This property makes it suitable for state estimation, where the output of a target is referred to as a noise-free interpretation of the input signal. Hence, bearings-only passive state estimation of an underwater moving target is a purely nonlinear problem in which the dynamics of a target are continuously time-varying.

In our proposed study, we model NARX neural intelligence to approximate the current state of the underwater dynamic object in different ocean environments. Our key objective in this study is to design an appropriate framework to investigate the time series statistics of passive noisy measurements from the passive target. This proposed work establishes a novel state estimation application of neural networks to analyze different underwater scenarios for semi-curved trajectory of the target. The simulation results are compared with our recent reported study [21], in which spherical radial cubature Kalman filter (SRCKF) and UKF are used for the same state estimation model.

**Figure 1:**
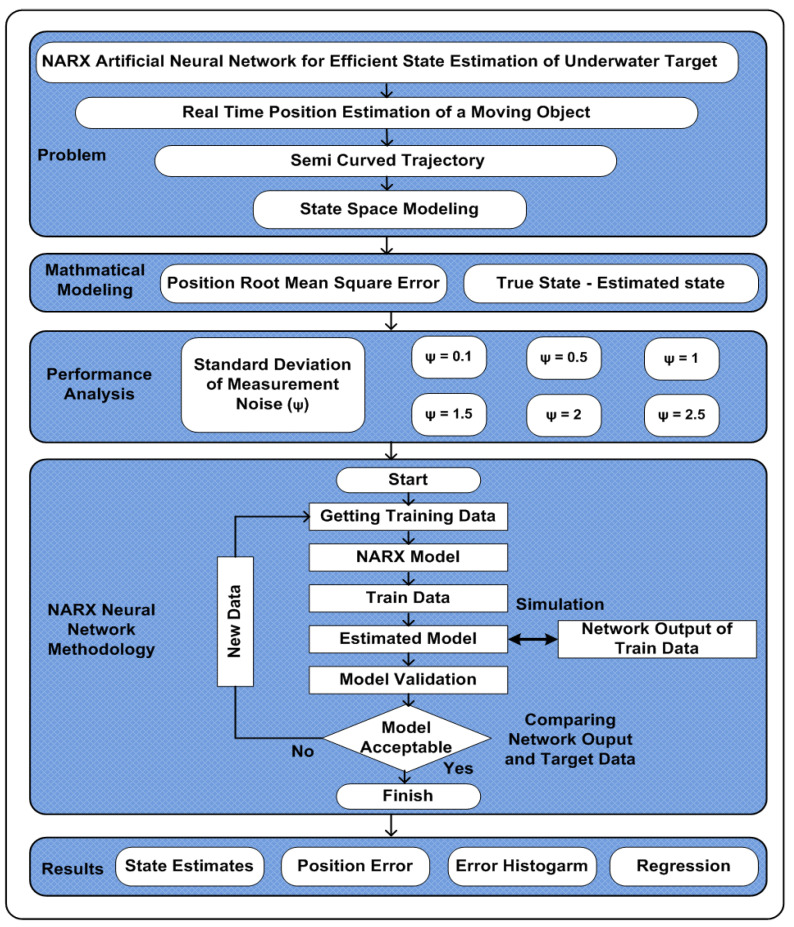
Flow chart of the state estimation scheme.

The numerical values of white Gaussian measurement noise are varied to assume cluttered and perfect underwater environment. Therefore, in the proposed study, NARX neural computing is investigated for position RMSE among actual and predicted location of an underwater kinetic vehicle. A comprehensive flow chart of the proposed work is illustrated in Figure 1. The highlights of the given scheme are summarized as follows:

- A performance based intelligent computing is presented for the accurate location estimation of a passive underwater target by manipulating the capability of NARX based neural network.
- Wiener Velocity Motion (WVM) model is exploited for designing the dynamics of target in semi-curved trajectory with the parameter of standard deviation of measurement noise.
- State estimates and position error for passive target prove the worth of proposed intelligent computing over conventional nonlinear variants of KF based on SRCKF and UKF.
- The performance is further endorsed through minimum RMSE criterion in terms of accuracy and better convergence rate than conventional nonlinear KFs.

The rest of the study is organized as follows—Section 2 initiate the passive state estimation system modeling based on state-space phenomena. Then, in Section 3 the methodology and architecture of NARX neural intelligence is presented with the procedure of training, testing and validation. Section 4 covers the experimentation of neural network and its detailed simulation results. The conclusion and a proposal for future study are revealed in Section 5.

## 2. Passive State Estimation System Model

**Figure 2:**
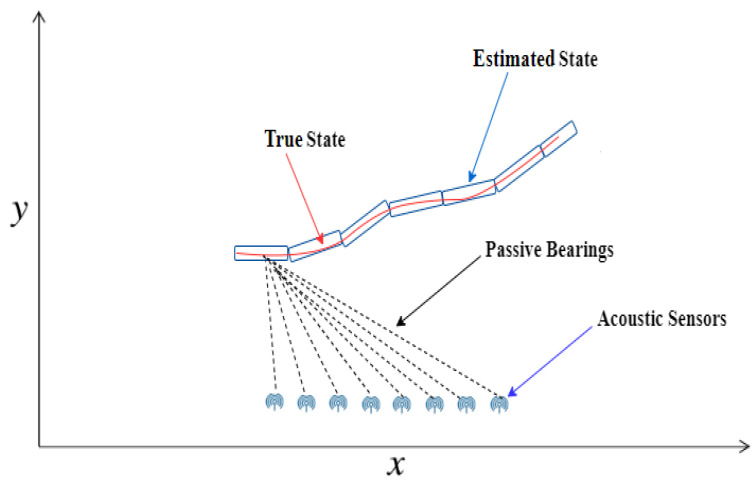
Passive state estimation architecture.

In this portion of the study, a passive bearings-only state estimation system model is designed by following the state-space methodology. The kinetics of an underwater dynamic object are designed in a two-dimensional Cartesian coordinates system for precise real-time position estimation. There are eight acoustic sensors for collecting and combining passive bearings emitted from target. The passive bearings collected from acoustic sensors depend upon the horizontal angle and orientation of each array component. In the given system model, movement of the target is assumed in a continuous real semi-curved trajectory, which we aim to approximate with NARX neural network. This passive state estimation architecture is shown in Figure 2.

### 2.1. Dynamic Model

In the dynamic model, target state vector consist on position $\left(x_{n},y_{n}\right)$ and velocity $\left(dx_{n},dy_{n}\right)$ at time step n in the two-dimensional rectangular coordinate system [21]. These motion variables are illustrated in state vector of the target $S_{n}^{t}$ as:

(1) $$S_{n}^{t}={\left[\begin{array}{cccc} x_{n}^{t} & y_{n}^{t} & dx_{n}^{t} & dy_{n}^{t} \end{array}\right]}^{T},$$

where ${\left[.\right]}^{T}$ is representing transpose of matrix in the above equation. Also, at the observer platform the state vector is represented as:

(2) $$S_{n}^{b}={\left[\begin{array}{cccc} x_{n}^{b} & y_{n}^{b} & dx_{n}^{b} & dy_{n}^{b} \end{array}\right]}^{T}.$$

A comparative state vector of target and observer is defined with the following relationship as:

(3) $$S_{n}=S_{n}^{t}-S_{n}^{b}={\left[\begin{array}{cccc} x_{n} & y_{n} & dx_{n} & dy_{n} \end{array}\right]}^{T}.$$

In this study, kinetics of underwater dynamic passive target are associated with discrete-time WVM model. Thus, the dynamic model based on the state vector is defined as:

(4) $$S_{n}=F_{n-1}S_{n-1}+\upsilon_{n-1},$$

meanwhile state conversion matrix in the above equation is represented with $F_{n-1}$, which is used to analyze the response of the given model. Independent white Gaussian process noise of zero mean is denoted with $\upsilon_{n-1}$ in the above model. Sampling interval di for process noise is given as:

(5) $$di=[i_{n-1}-i_{n}],$$

whereas the state conversion matrix $F_{n-1}$ is described as:

(6) $$F_{n-1}=\left[\begin{array}{cccc} 1 & 0 & di & 0\\ 0 & 1 & 0 & di\\ 0 & 0 & 1 & 0\\ 0 & 0 & 0 & 1 \end{array}\right].$$

The dynamic model given in Equation (4) should be in discrete-time for accurate state estimation with NARX neural model. The discrete dynamic model equation will be applied for evaluating the system for time steps n, while this time step is multiple of di. The discrete-time dynamic model with appropriate values of its parameters is expressed as:

(7) $$S_{n}=\underset{F_{n-1}}{\underbrace{\left[\begin{array}{cccc} 1 & 0 & di & 0\\ 0 & 1 & 0 & di\\ 0 & 0 & 1 & 0\\ 0 & 0 & 0 & 1 \end{array}\right]}}\underset{S_{n-1}}{\underbrace{\left[\begin{array}{c} x_{n-1}\\ y_{n-1}\\ dx_{n-1}\\ dy_{n-1} \end{array}\right]}}+\upsilon_{n-1}.$$

Process noise $\upsilon_{n-1}$ in the above discrete-time dynamic model can be calculated with its covariance $C_{n-1}^{d}$ as:

(8) $$\upsilon_{n-1}\approx {N(0,C}_{n-1}^{d}),$$

meanwhile above equation can be written as:

(9) $$C_{n-1}^{d}=E\left[\upsilon_{n-1}\upsilon_{n-1}^{T}\right].$$

Likewise, dynamic model, the covariance of process noise should also be given in discrete form with spectral density ϖ as:

(10) $$C_{n-1}^{d}=\left[\begin{array}{cccc} \frac{1}{3}di^{3} & 0 & \frac{1}{2}di^{2} & 0\\ 0 & \frac{1}{3}di^{3} & 0 & \frac{1}{2}di^{2}\\ \frac{1}{2}di^{2} & 0 & di & 0\\ 0 & \frac{1}{2}di^{2} & 0 & di \end{array}\right]\varpi .$$

### 2.2. Measurement Model

Moreover, state-space methodology is also associated with the measurement model [19]. At time step n the measurement model is designed as:

(11) $$M_{n}=h\left(S_{n},\Gamma_{n}\right).$$

In the above model, measurement function h(.) depends upon current noisy passive measurements from acoustic sensors at time step n. Independent white Gaussian measurement noise in the above measurement model is denoted with $\Gamma_{n}$. Passive bearings $\theta_{n}$ impinging at array element z and current position of the dynamic object $\left(x_{n},y_{n}\right)$ at time step n are combined in measurement function as:

(12) $$h\left(S_{n}\right)=\theta_{n}=\left[\frac{y_{n}-\alpha_{y}^{z}}{x_{n}-\alpha_{x}^{z}}\right].$$

Based upon measurement function and measurement noise the inclusive measurement model $M_{n}^{z}$ at time step n for antenna element z is expressed as:

(13) $$M_{n}^{z}=\underset{\theta_{n}}{\underbrace{\tan^{-1}\left[\frac{y_{n}-\alpha_{y}^{z}}{x_{n}-\alpha_{x}^{z}}\right]}}+\Gamma_{n}^{z}.$$

In measurement model $\alpha_{y}^{z},\alpha_{x}^{z}$ are representing the localization function of sensors z in two-dimensional rectangular coordinates, while independent measurement noise $\Gamma_{n}^{z}$ have zero mean and covariance $C_{n-1}^{m}$. The covariance of measurement noise can be written as:

(14) $$\Gamma_{n}^{z}\approx N\left(0,C_{n-1}^{m}\right),$$

whereas

(15) $$C_{n-1}^{m}=\mathrm{diag}(\Psi_{M}^{2}).$$

In the above covariance equation, Ψ is representing the standard deviation of measurement noise which have larger impact in given passive state estimation scheme. In the proposed study, the standard deviation of measurement noise Ψ is oscillated for designing ideal and cluttered noisy scenarios in ocean environment. In simulations, standard deviation of measurement noise is chosen as a criterion for analyzing the accuracy of neural scheme and nonlinear KFs. The starting location of concern target is assumed at $S_{0}$ = ${\left[-2\;-0.5\;1\;0\right]}^{T}$. The prior distribution for the initial state is $S_{0}\approx N(0,P_{0})$ and $P_{0}$ is assumed as:

(16) $$P_{0}=\left[\begin{array}{ccccc} 0.1 & 0 & \cdots & 0 & 0\\ 0 & 0.1 & \cdots & 0 & 0\\ \vdots & \vdots & \ddots & 0 & 0\\ 0 & 0 & 0 & 10 & 0\\ 0 & 0 & 0 & 0 & 10. \end{array}\right].$$

## 3. Artificial Neural Network (ANN) Modeling

In this section of the study, ANN modeling is briefly explained with a systematic description. ANNs, simply known as neural networks, are actually intelligent computing frameworks developed by the biological neural systems that integrate animal brains. A typical ANN is constructed from a set of bound nodes called artificial neurons. Each bound node is used to propagate signals between neurons. In our proposed study, NARX based ANN is designed for the accurate state prediction of an underwater target. Detailed modeling of NARX with its training method is explained in the below subsections.

### 3.1. Nonlinear Autoregressive with Exogenous Input (NARX) Model

**Figure 3:**
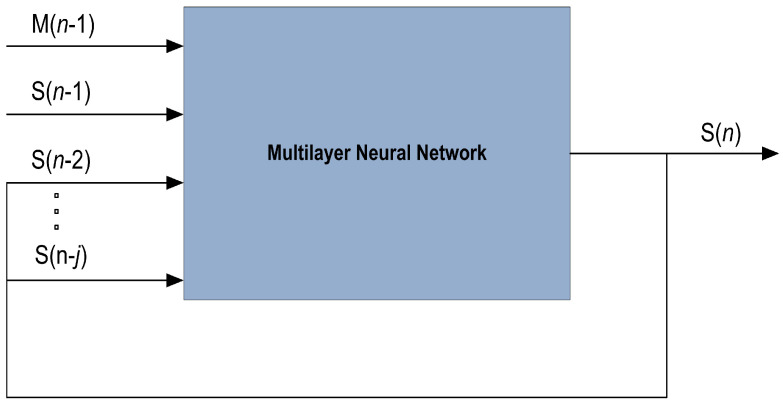
Multilayer structure of NARX neural network.

Here, the methodology of NARX neural intelligence is mathematically designed for state estimation of the underwater moving object. The importance of relationship among designed time series data and supplementary external data in real-life applications cannot be ignored. A common phenomenon in state estimation problems is that the performance of the estimation algorithm heavily depends upon noisy bearings. Therefore, the previous knowledge of noisy bearings can be handy for the modeling of time series to obtain better state estimation performance. A multilayer structure of NARX neural network is depicted in Figure 3.

In the methodology of NARX, neural learning is applied for predicting future values in time series by efficiently incorporating previous data. External input and latter outputs of time series data are responsible for estimating the next value in nonlinear model of the NARX neural network. The multilayer structure of the NARX is designed with hidden layer, delay layer, input layer and output layer. Here we want to accurately predict state vector series S(n) of passive object for j previous values of real data series S with additional input of measurement series M(n) having input delay of k. Based on these notations behavior of NARX hidden nodes for predicting time series is modeled as:

(17) $$S\left(n\right)=R\left(\begin{array}{c} M(n-1),\;M(n-2),\;\ldots ,M(n-k)\\ S(n-1),\;S(n-2),\;\ldots ,S(n-j) \end{array}\right)+e\left(n\right).$$

In the above nonlinear model of the NARX neural scheme [34], measurements M(n) is a Q dimension input vector while S(n) is representing output vector having R dimension. k and j are input and output delay orders correspondingly, whereas e(n) is showing neural network error.

(18) $$y\left(n\right)=g_{v_{1}}\left(M\left(n\right)u_{v_{1}}+a_{v_{1}}\right),$$

(19) $$S\left(n\right)=g_{v_{2}}\left(y\left(n\right)u_{v_{2}}+a_{v_{2}}\right).$$

In Equation (18) hidden layer vector is denoted with y(n) having dimensions of v. Thresholds of hidden layer and input layer are represented with $a_{v_{1}}$ and $a_{v_{2}}$ accordingly. Weighted connections between hidden, delay and output layers are $u_{v_{1}}$ and $u_{v_{2}}$ respectively. $g_{v_{1}}$ is denoting the transfer function of hidden nodes while $g_{v_{2}}$ is representing the activation function of output nodes.

### 3.2. NARX Neural Network Architecture

NARX is a recursive discrete-time neural network consist on time series [44] and can be designed as:

(20) $$S\left(w+1\right)=R\left(\begin{array}{c} S(w),\;S(w-1),\;\ldots ,S(w-d,+1)\\ M(w-n+1),\;\;\ldots ,M(w-d,-n+1) \end{array}\right).$$

Assuming process dead time delay term n in the above model is approaching to zero and following this updated NARX, the neural model can be written as:

(21) $$S\left(w+1\right)=R\left(\begin{array}{c} S(w),\;S(w-1),\;\ldots ,S(w-d,+1)\\ M(w),M(w+1),\;\;\ldots ,M(w-d,1) \end{array}\right).$$

**Figure 4:**
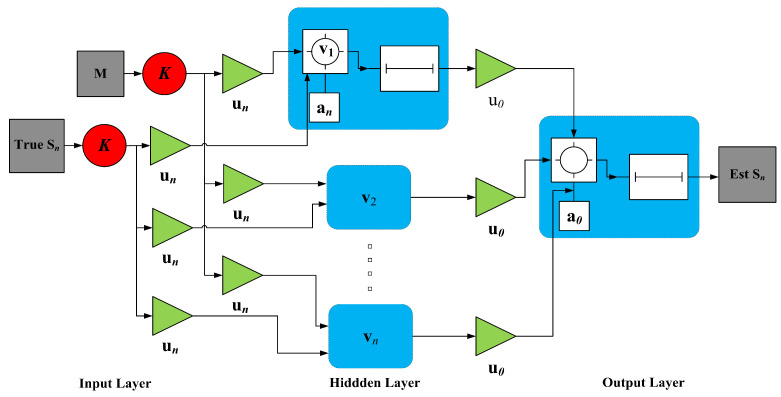
Architecture of NARX neural network.

The vector form of the above model is expressed as:

(22) S(w+1)=R(S(w),M(w)),

where input and output regresses are represented with vectors M(w) and S(w) correspondingly in the above vector form. Here we applied the strength of Levenberg–Marquardt (LM) algorithm to train neurons of NARX model. Architecture of the NARX neural network is shown in Figure 4.

### 3.3. Levenberg–Marquardt (LM) Training Method

The LM algorithm is widely applied in the literature as the fastest learning mechanism for the NARX neural network. This algorithm is formulated to compute derivatives of second-order without calculating the Hessian matrix so its training speed is increased. Two researchers Kenneth Levenberg and Donald Marquardt introduced LM training technique by integrating Gauss-Newton algorithm (GNM) with steepest descent method (SDM). The working principle of LM training scheme is joint training procedure of neurons by initially quadratic estimation is conducted with SDM and after that weights are updated and improved with GNM. Minimum of function L(M) is initially computed in the training algorithm from nonlinear functions as:

(23) $$L\left(M\right)=\sum_{b=1}^{c}\frac{{\left[l_{b}\left(M\right)\right]}^{2}}{2}.$$

Like the above model when sum of squares are combined in performance function, then Hessian matrix H and gradient χ can be calculated in the following form as:

(24) $$H=Z_{n}^{T}Z_{n},$$

(25) $$\chi =Z_{n}^{T}e_{n}.$$

In the Hessian matrix and gradient equation, Z represents the Jacobian matrix. First-order derivatives of neural network error are combined in this Jacobian matrix in correspondence with biases and weights, while in all training data points $e_{n}$ is representing neural network error vector. LM training technique involves the Jacobian matrix for computations purpose by incorporating the mean of the sum of the squared errors are combined in the performance function. The search space of LM algorithm is represented by the following equation as:

(26) $$\left(Z_{n}^{T}Z_{n}+\beta_{n}I\right)p_{n}=-Z_{n}^{T}l_{n}.$$

In the above search space equation, I is denoting the identity matrix while positive scalars are shown with $\beta_{n}$. Thus, in LM training method the rule of updating weights $u_{n}$ is expressed as:

(27) $$u_{n+1}=u_{n}-\frac{Z_{n}e_{n}}{\left(Z_{n}^{T}Z_{n}+\beta_{n}I\right)}.$$

### 3.4. Performance Evaluation Criterion

RMSE among true position and predicted position from state estimation techniques at each time instant n is taken as an evaluation criterion for analyzing the performance. The minimum value of RMSE is showing better accuracy of each algorithm. Position error equation in the form of RMSE is computed with the following relationship as:

(28) $$\mathrm{RMSE}(n)=\sqrt{\frac{1}{N}\sum_{n=1}^{N}{\left|S_{n}^{True}-\left.S_{n}^{Est}\right|\right.}^{2}}.$$

The actual state vector of the moving target in the above fitness function is symbolized with $S_{n}^{True}$, whereas the predicted state of object from the proposed algorithm NARX neural network and conventional Kalman filtering techniques is denoted by $S_{n}^{Est}$. The maximum number of samples or data symbols is 500, which are shown with N in above RMSE function. In the semi curved path at each point n, the position error is computed in this study for performance analysis.

## 4. Simulation Results and Discussion

**Figure 5:**
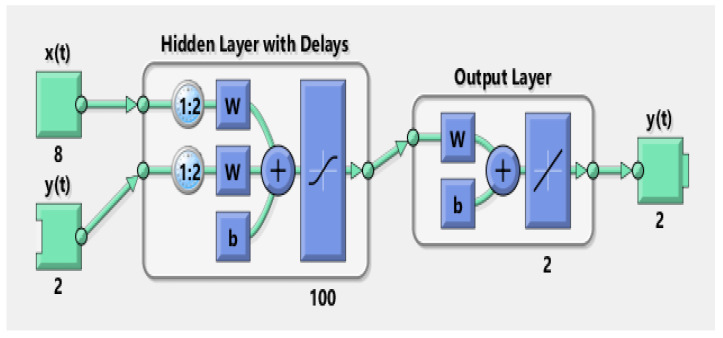
Toolbox structure of NARX Model for state estimation.

Here in the simulation results and discussion section, the state estimates, position error, error histogram and regression analysis of proposed NARX neural scheme are comprehensively explained. Arithmetical values of measurement noise are accelerated from 0.1 to 2.5 radian for creating six different scenarios in the ocean environment. The least value of measurement noise Ψ = 0.1 radian is showing an ideal underwater atmosphere, while the maximum value of measurement noise Ψ = 2.5 radian is representing the complex noisy ocean environment. Both measurements from the sensor network and true state of the dynamic object are used as input of NARX neural model to accurately find the estimated state of the dynamic target. The structure of NARX neural model is simulated in the neural network toolbox of MATLAB. This structure consists of one input, one hidden and one output layer with feedback path as revealed in Figure 5. True state of passive target is considered as one input value in NARX model to enhance prediction output. In hidden layer, the activation function of neurons is based on sigmoid function. In simulations of NARX structure, the hidden layer is based upon 100 hidden neurons for better state estimation results.

LM algorithm is used to train NARX neural network in epoch form by following the Back-propagation algorithm through time phenomena. The network is trained using the in epoch wise mode. In the designing procedure, passive bearings collected at the acoustic sensors and true position data are used for estimation and evaluation of results. In overall time series, 70% data is used for training, validation is using 15% data and the remaining 15% data is used for testing purposes of the results. In this study, the LM algorithm is used to train the neural network. In the below figure MATLAB toolbox structure of NARX is given in which we give passive measurements M(n) at the input of x(t), while the target state from true trajectory S(n) is given at y(t) input.

State estimation framework consists of various parameters that need to be properly tuned for obtaining better performance from state prediction algorithms. The parameters and their relevent setting is selected on a similar manner as in reported studies [3,19,21,22]. Appropriate values of these parameters are listed in Table 1.

### 4.1. State Prediction of Passive Target by Varying Standard Deviation of Measurement Noise

**Figure 6:**
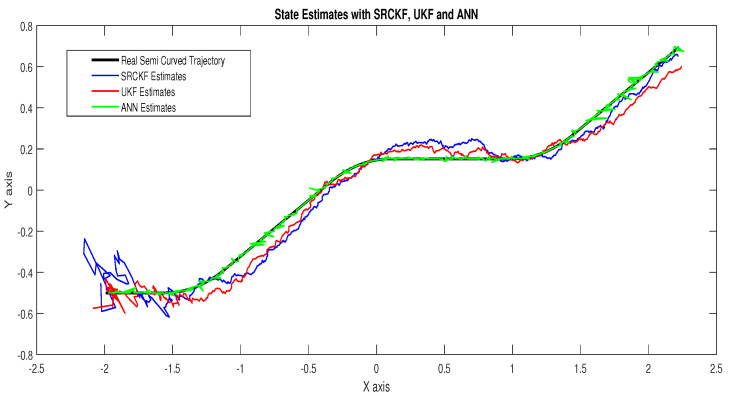
State estimates of passive target.

Here, MATLAB simulation results of SRCKF, UKF and ANN are conducted for estimating the real-time state of the passive moving target in rectangular coordinates. The performance of ANN for efficient state estimation is analyzed over six different scenarios of standard deviation of measured noise. These scenarios represent the ideal and noisy underwater environment in which the estimation capability of ANN is exploited. For each scenario, performance analysis of filtering and NARX neural network can be seen from Figure 6 to Figure 23 in the form of state estimates, position error, error histogram and regression of actual and estimated trajectory. Here six different variations of measurement noise are represented with their mathematical equations and simulations results as:

#### 4.1.1. Scenario 1: Measurement Noise = 0.1 Radian

In this scenario, standard deviation of measurement noise is taken as Ψ = 0.1 radian which is showing a smooth ocean atmosphere. Whereas the covariance of measured noise is calculated from this value of standard deviation of measurement noise as:

(29) $$C_{n-1}^{m}=\mathrm{diag}(\Psi_{M}^{2}).$$

Covariance in this scenario is developing independent white Gaussian measurement noise for z sensors at time step n as:

(30) $$\Gamma_{n}^{z}\approx N\left(0,C_{n-1}^{m}\right).$$

Above computed independent white Gaussian measurement noise is adding in measurement model as:

(31) $$M_{n}^{z}=\underset{\theta_{n}}{\underbrace{\tan^{-1}\left[\frac{y_{n}-\alpha_{y}^{z}}{x_{n}-\alpha_{x}^{z}}\right]}}+\Gamma_{n}^{z}.$$

The overall measurement model M for sensor z at time step n is described above in which passive measurements from eight sensors are combined with independent white Gaussian measurement noise. These passive measurements of sensors computed in the above model are applied to NARX neural network as input time series $M_{n}^{z}$. Target’s time series which is defining desired output is taken from state vector of the dynamic model as:

(32) $$S_{n}=S_{n}^{t}-S_{n}^{b}={\left[\begin{array}{cccc} x_{n} & y_{n} & dx_{n} & dy_{n} \end{array}\right]}^{T}.$$

In the above, state vector $x_{n}$ and $y_{n}$ are defining the position of object in x-y axis correspondingly at time step n. These real positions of the dynamic passive object are designed for complete semi-curved trajectory and applied in NARX neural network modeling as target time series for approximating desired current states of the object. Simulations results in the form of state estimates, location error, error histogram and regression of actual and estimated trajectory for this scenario are shown below.

**Figure 7:**
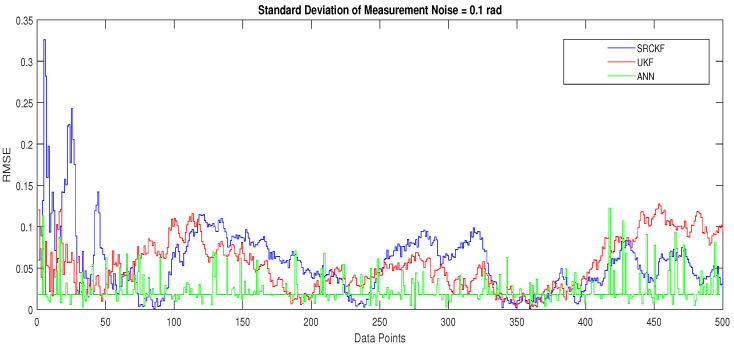
Position error between true and estimated trajectory.

**Figure 8:**
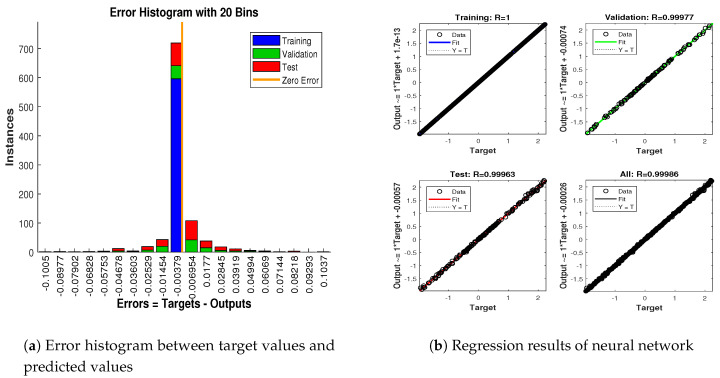
Error histogram and regression analysis of ANN in scenario 1.

In scenario 1, an ideal underwater environment is taken by assuming the lowest value of the standard deviation of measured noise. The major observations in this scenario are explained as:

- In Figure 6 state estimates of ANN are compared with other two conventional filtering techniques and it is clear that ANN is exactly following the true trajectory of the dynamic object with respect to other techniques.
- In Figure 7 RMSE between true and estimated position of dynamic object is represented which is clearly showing that ANN is performing far better from conventional filtering algorithms of SRCKF and UKF.
- In Figure 8a, the error histogram is shown between target values S(n),S(n−1),...,S(n−j) and the estimated value $S_{n}^{Est}$ of target’s position after training feedback ANN. As these error values indicate how predicted values differ from the target value and it can be negative. The total error of neural network is distributed in 20 smaller vertical bars in the graph of Figure 8a, which are called bins. In a specific bin, the number of data points from the total data set are shown on the Y-axis. IN the middle of the plot, a bin corresponding to the error of −0.00379 and the height of that bin for training data set lies near to 700 while validation and test data set lie between 600 and 700. This means that many samples from different data sets have an error lies in that following range. The zero error line corresponds to the zero-error value on the error axis (i.e., X-axis). In this case, the zero error point falls under the bin with center −0.00379.
- In Figure 8b, the regression results of the ANN scheme are presented for the training, validation and testing process. The overall data set is divided into these three with a ratio of 75%, 15% and 15% correspondingly. Neural network analysis of regression is actually a combination of statistical procedures for predicting correspondence among single output variable $S_{n}^{Est}$ and target variable $S_{n}$. In the regression results, it can be seen that the actual target and predicted output are overlapping each other. An almost linear behavior among target and output values is observed, which is clear evidence for the effectiveness of the proposed NARX model.

#### 4.1.2. Scenario 2: Measurement Noise = 0.5 Radian

In scenario 2, standard deviation of measurement noise is taken as Ψ = 0.5 radian, which produces a small amount of noisy bearings at acoustic sensors. While the covariance of measurement noise from this value of standard deviation of measurement noise is given as:

(33) $$C_{n-1}^{m}=\mathrm{diag}(\Psi_{M}^{2}).$$

Covariance in this above equation is based on independent white Gaussian measurement noise for z sensors at time step n as:

(34) $$\Gamma_{n}^{z}\approx N\left(0,C_{n-1}^{m}\right).$$

Above computed independent white Gaussian measurement noise is adding in measurement model as:

(35) $$M_{n}^{z}=\underset{\theta_{n}}{\underbrace{\tan^{-1}\left[\frac{y_{n}-\alpha_{y}^{z}}{x_{n}-\alpha_{x}^{z}}\right]}}+\Gamma_{n}^{z}.$$

Total passive measurements M combining from acoustic sensor z at time step n are described in the above measurement model with a measurement noise of 0.5 radian. These passive measurements computed in the above model are applied to NARX neural network as input time series $M_{n}^{z}$. Target time series is taken from the state vector of the dynamic model as:

(36) $$S_{n}=S_{n}^{t}-S_{n}^{b}={\left[\begin{array}{cccc} x_{n} & y_{n} & dx_{n} & dy_{n} \end{array}\right]}^{T}.$$

**Figure 9:**
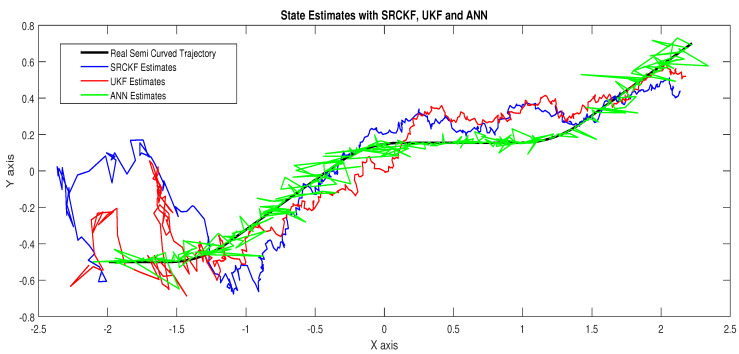
State estimates of passive target.

**Figure 10:**
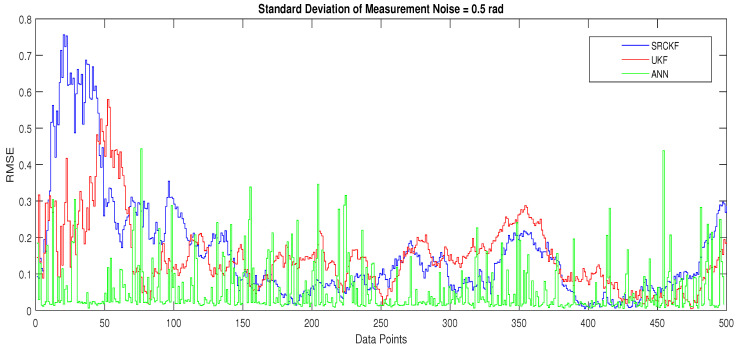
Position error between true and estimated trajectory.

**Figure 11:**
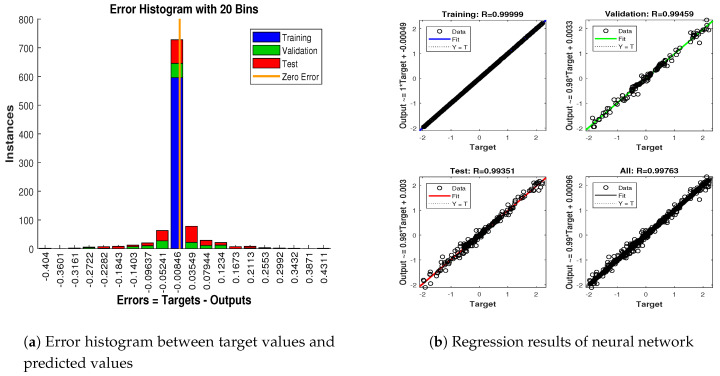
Error histogram and regression of ANN in scenario 2.

These real positions of the dynamic passive object are designed for complete semi-curved trajectory and applied in NARX neural network modeling as target time series for estimating desired real-time states of the object. Simulations results in the form of state estimates, position error, error histogram and regression results of actual and estimated trajectory for this scenario are shown in Figure 9, Figure 10 and Figure 11.

The explanation of all simulation results is given as:

- In Figure 9, state estimates of ANN are compared with other two conventional filtering techniques and it is clear that ANN is also showing better accuracy rate for estimating the true trajectory of the dynamic object in this scenario.
- In Figure 10, RMSE between true and estimated position of the dynamic object is represented which is expressing competency of ANN over conventional filtering algorithms of SRCKF and UKF.
- In Figure 11a, error histogram is shown between target values S(n),S(n−1),...,S(n−j) and estimated value $S_{n}^{Est}$ of the target’s position after training feedback ANN. At the mid of the plot, a bin corresponding to the error of −0.00846 and the height of that bin for training data set lies near to 700 samples while validation and test data set lie between 700 and 800 samples. It means that many samples from different data sets have an error lies in that following range. In this scenario, zero error point falls under the bin with center −0.00846.
- In Figure 11b, the regression results of ANN scheme are presented for training, validation and testing process. In regression results efficiency of ANN is seen by linear trend and the adjacent response of actual target and predicted output.

#### 4.1.3. Scenario 3: Measurement Noise = 1 Radian

In this scenario, the standard deviation of measurement noise is taken as Ψ = 1 radian which depicts that there is sufficient increment in noise. While the covariance of the measurement noise for this value of standard deviation is computed as:

(37) $$C_{n-1}^{m}=\mathrm{diag}(\Psi_{M}^{2}).$$

Covariance in this scenario depends upon independent white Gaussian measurement noise for z sensors at time step n as:

(38) $$\Gamma_{n}^{z}\approx N\left(0,C_{n-1}^{m}\right).$$

Above computed independent white Gaussian measurement noise is adding in measurement model as:

(39) $$M_{n}^{z}=\underset{\theta_{n}}{\underbrace{\tan^{-1}\left[\frac{y_{n}-\alpha_{y}^{z}}{x_{n}-\alpha_{x}^{z}}\right]}}+\Gamma_{n}^{z}.$$

The overall measurement model M for sensor z at time step n is described above in which passive measurements from eight acoustic sensors are combining with independent white Gaussian measurement noise assumed in this scenario. These passive measurements of sensors computed in the above model are applied to NARX neural network as input time series $M_{n}^{z}$. Target time series is taken from state vector of the dynamic model as:

(40) $$S_{n}=S_{n}^{t}-S_{n}^{b}={\left[\begin{array}{cccc} x_{n} & y_{n} & dx_{n} & dy_{n} \end{array}\right]}^{T}.$$

Real positions of the dynamic passive object are modeled for complete semi-curved trajectory and applied in NARX neural network modeling as target time series for estimating desired real-time states of the object. Simulation results for this scenario in the form of state estimates, least position error, error histogram and regression analysis of actual and estimated trajectory are shown below.

**Figure 12:**
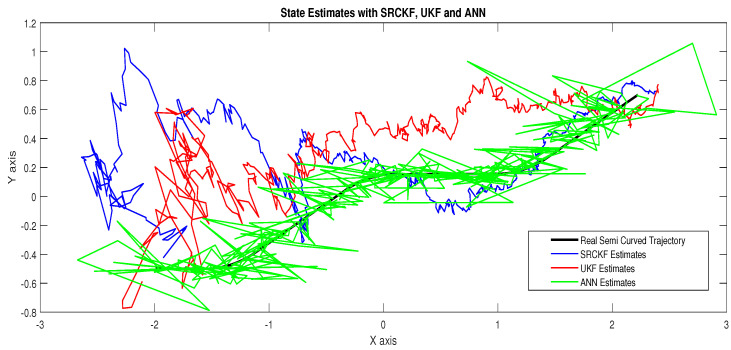
State estimates of passive target.

**Figure 13:**
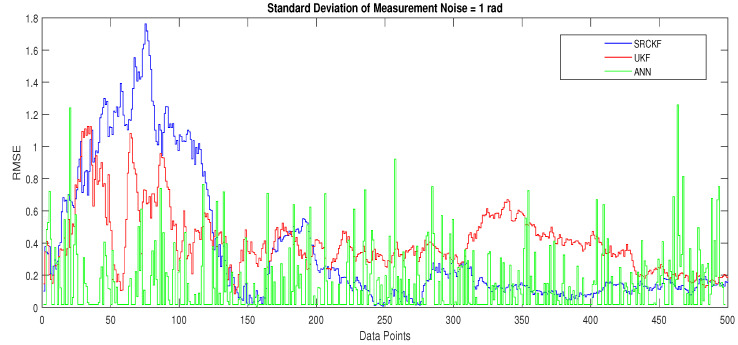
Position error between true and estimated trajectory.

**Figure 14:**
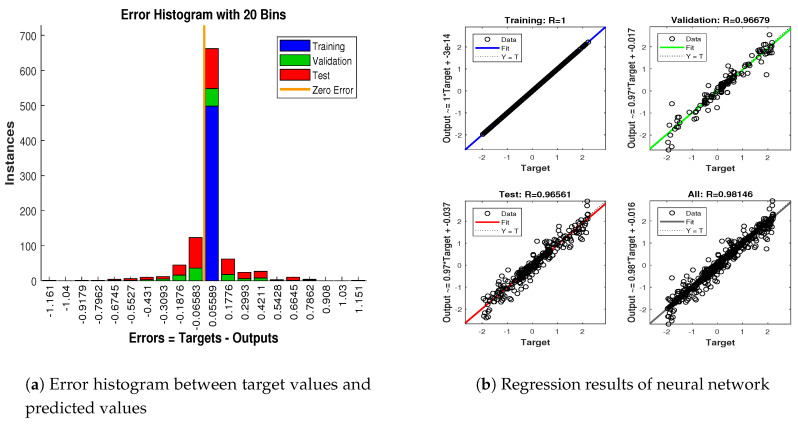
Error histogram and regression of ANN in scenario 3.

Simulation results in this scenario are explained as:

- In Figure 12, the state estimates of all techniques are shown in which it is wort noting that again ANN is estimating real state object with efficiency as compared to SRCKF and UKF.
- In Figure 13, RMSE between true and estimated position of the dynamic object is represented which is also depicting the competency of ANN as compared to conventional filtering algorithms.
- In Figure 14a, error histogram is shown between target values S(n),S(n−1),...,S(n−j) and the estimated value $S_{n}^{Est}$ of target’s position after training feedback ANN. At the mid of the plot, a bin corresponding to the error of 0.05589 and the height of that bin for training data set lie near to 700 data points while validation and test data set lies between 600 and 700 data points. It means that many samples from different data sets have an error that lies in that given range. In this scenario, the zero error point falls under the bin with center 0.05589.
- In Figure 14b, regression results of ANN scheme are presented for training, validation and testing process. In regression results, there is small divergence between actual target and predicted output because of sufficient amount of noise.

#### 4.1.4. Scenario 4: Measurement Noise = 1.5 Radian

In this scenario, the standard deviation of measurement noise is taken as Ψ = 1.5 radian, which shows a relatively noisy underwater environment. While the covariance of measurement noise computed in this scenario is defined with the following mathematical expression as:

(41) $$C_{n-1}^{m}=\mathrm{diag}(\Psi_{M}^{2}).$$

Covariance is depending on independent white Gaussian measurement noise for z sensors at time step n as:

(42) $$\Gamma_{n}^{z}\approx N\left(0,C_{n-1}^{m}\right).$$

Above computed independent white Gaussian measurement noise is adding in measurement model as:

(43) $$M_{n}^{z}=\underset{\theta_{n}}{\underbrace{\tan^{-1}\left[\frac{y_{n}-\alpha_{y}^{z}}{x_{n}-\alpha_{x}^{z}}\right]}}+\Gamma_{n}^{z}.$$

Measurement model M for sensor z at time step n is designed above in which passive measurements from acoustic array elements are combining with independent white Gaussian measurement noise. These passive bearings are applied to NARX neural network as input time series $M_{n}^{z}$. Target time series is taken from state vector of the dynamic model as:

(44) $$S_{n}=S_{n}^{t}-S_{n}^{b}={\left[\begin{array}{cccc} x_{n} & y_{n} & dx_{n} & dy_{n} \end{array}\right]}^{T}.$$

**Figure 15:**
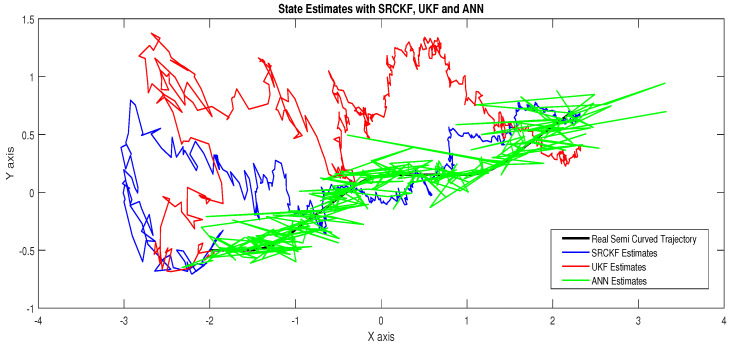
State estimates of passive target.

**Figure 16:**
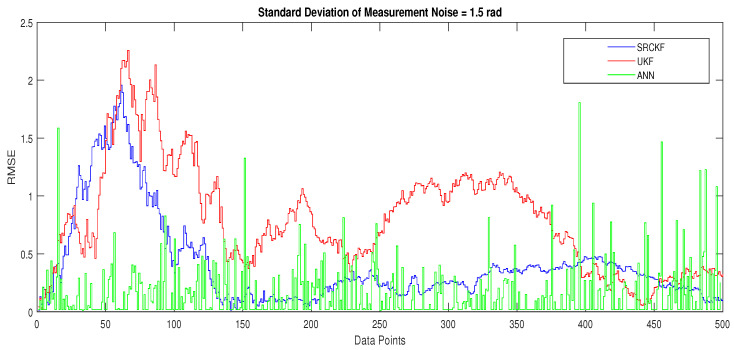
Position error between true and estimated trajectory.

**Figure 17:**
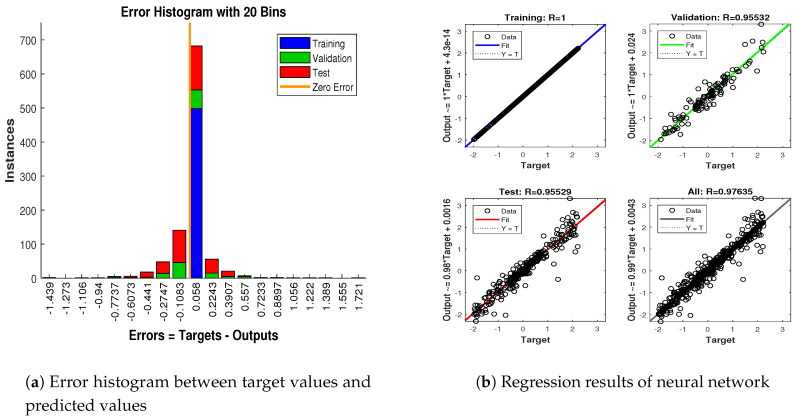
Error histogram and regression of ANN in scenario 4.

$x_{n}$ and $y_{n}$ in above state vector are defining the position of object in x-y axis correspondingly at time step n. These real positions of the dynamic passive object are designed for complete semi-curved trajectory and applied in NARX neural network modeling as target time series for estimating desired real-time state of the object. Simulations results in the form of state estimates, position error, error histogram and regression analysis of actual and estimated trajectory for this scenario are shown in Figure 15, Figure 16 and Figure 17.

The major observations in this scenario are discussed as:

- In Figure 15, state estimates of ANN, SRCKF and UKF are presented in which it is clearly seen that ANN is converging more than other two algorithms.
- In Figure 16, RMSE between true and estimated position of the dynamic object is represented which is also showing that ANN has less position error from SRCKF and UKF.
- In Figure 17a, error histogram is shown between target values S(n),S(n−1),...,S(n−j) and estimated value $S_{n}^{Est}$ of target’s position after the training feedback ANN model. At the mid of the plot, a bin corresponding to the error of 0.058 and the height of that bin for training data set lies near to 700 samples while validation and test data set lie between 600 and 700 samples. It means that many samples from different data sets have an error lies in that following range. Zero error line corresponding to the zero-error value on the error axis. In this scenario, zero error point falls under the bin with center 0.058.
- In Figure 17b, the regression analysis of ANN modeling is presented for the training, validation and testing process. In regression results some distance is appearing between actual target and predicted output because of noisy ocean condition.

#### 4.1.5. Scenario 5: Measurement Noise = 2 Radian

In this scenario, standard deviation of measurement noise is taken as Ψ = 2 radian. While the covariance of measurement noise from this value of standard deviation of measurement noise is computed as:

(45) $$C_{n-1}^{m}=\mathrm{diag}(\Psi_{M}^{2}).$$

Covariance in this scenario develops from independent white Gaussian measurement noise for z sensors at time step n as:

(46) $$\Gamma_{n}^{z}\approx N\left(0,C_{n-1}^{m}\right).$$

The above computed independent white Gaussian measurement noise is adding up in measurement model as:

(47) $$M_{n}^{z}=\underset{\theta_{n}}{\underbrace{\tan^{-1}\left[\frac{y_{n}-\alpha_{y}^{z}}{x_{n}-\alpha_{x}^{z}}\right]}}+\Gamma_{n}^{z}.$$

Passive bearings collected at sensors are computed in the above model and applied to NARX neural network as input time series $M_{n}^{z}$. Target time series which is defining desire output is taken from state vector of the dynamic model as:

(48) $$S_{n}=S_{n}^{t}-S_{n}^{b}={\left[\begin{array}{cccc} x_{n} & y_{n} & dx_{n} & dy_{n} \end{array}\right]}^{T}.$$

These real-time positions of the dynamic passive object are designed for each point of complete semi-curved trajectory and applied in NARX neural network modeling as target time series for estimating desired real-time states of the object. Simulations results in the form of state estimates, position error, error histogram and regression analysis of actual and estimated trajectory for this scenario are shown below.

**Figure 18:**
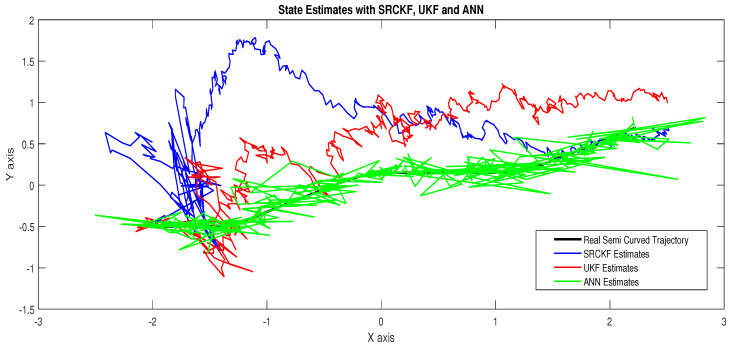
State estimates of passive target.

**Figure 19:**
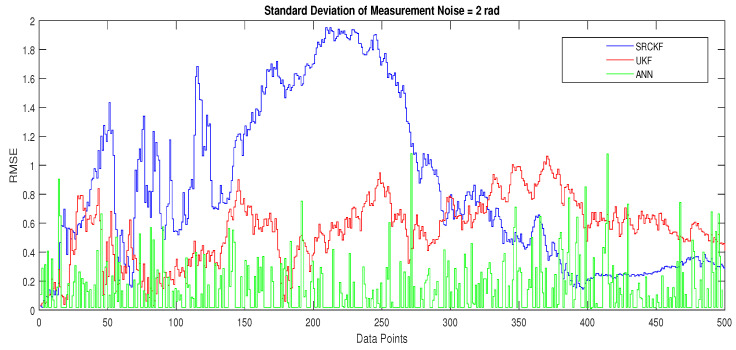
Position error between true and estimated trajectory.

**Figure 20:**
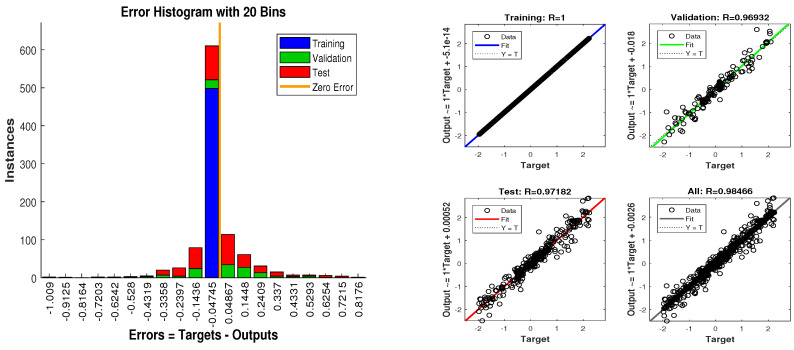
Error histogram and regression of ANN in scenario 5.

Concluding remarks on simulation results of this scenario are given as:

- State estimates of SRCKF, UKF and ANN are presented in Figure 18 and again ANN performs far better at estimating the semi-curved trajectory than the other two algorithms.
- In Figure 19, RMSE between true and estimated position of dynamic object is represented which is showing that ANN has less position error as compared SRCKF and UKF.
- In Figure 20a, the error histogram is shown between target values S(n),S(n−1),...,S(n−j) and estimated value $S_{n}^{Est}$ of target’s position after training feedback ANN. At the mid of the plot, a bin corresponding to the error of −0.04745 and the height of that bin for training data set lie near to 600 samples while validation and test data set lie between 500 and 600 samples. It means that many samples from different data sets have an error lies in that following range. In this case, the zero error point falls under the bin with center −0.04745.
- In Figure 20b, the regression results of ANN modeling are presented for the training, validation and testing process. In the regression results, some divergence between the actual target and the predicted output appears because of complex noisy bearings.

#### 4.1.6. Scenario 6: Measurement Noise = 2.5 Radian

In the last scenario of this study, the standard deviation of measurement noise is taken as Ψ = 2.5 radian, which shows an extremely noisy and cluttered ocean environment. The covariance of measurement noise calculated from this value of standard deviation of measurement noise is written as:

(49) $$C_{n-1}^{m}=\mathrm{diag}(\Psi_{M}^{2}).$$

Covariance is designed from independent white Gaussian measurement noise for z sensors at time step n as:

(50) $$\Gamma_{n}^{z}\approx N\left(0,C_{n-1}^{m}\right).$$

Above computed independent white Gaussian measurement noise is adding in measurement model as:

(51) $$M_{n}^{z}=\underset{\theta_{n}}{\underbrace{\tan^{-1}\left[\frac{y_{n}-\alpha_{y}^{z}}{x_{n}-\alpha_{x}^{z}}\right]}}+\Gamma_{n}^{z}.$$

Measurement model M for sensor z at time step n is presented above in which passive measurements from eight acoustic sensors are combined with independent white Gaussian measurement noise. These passive measurements of sensors computed in the above model are applied to the NARX neural network as input time series $M_{n}^{z}$. The target time series, which defines the required output is taken from the state vector of the dynamic model as:

(52) $$S_{n}=S_{n}^{t}-S_{n}^{b}={\left[\begin{array}{cccc} x_{n} & y_{n} & dx_{n} & dy_{n} \end{array}\right]}^{T}.$$

These real positions of the dynamic passive object shown in the above equation are designed for complete semi-curved trajectory and applied in NARX neural network modeling as a target time series for estimating the desired real-time states of object. In this scenario, the simulation results in the form of state estimates, position error, error histogram and regression results of actual and estimated trajectory are shown below.

**Figure 21:**
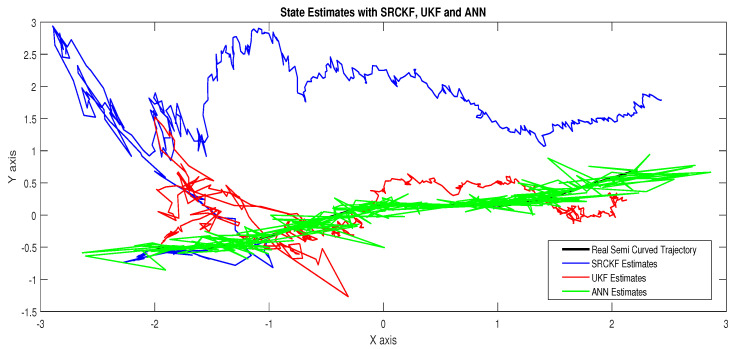
State estimates of passive target.

**Figure 22:**
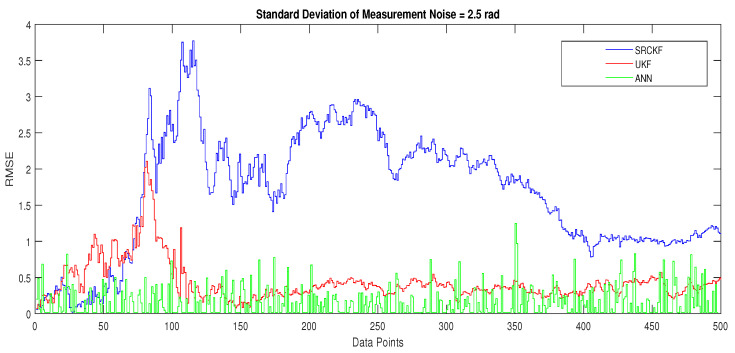
Position error between true and estimated trajectory.

**Figure 23:**
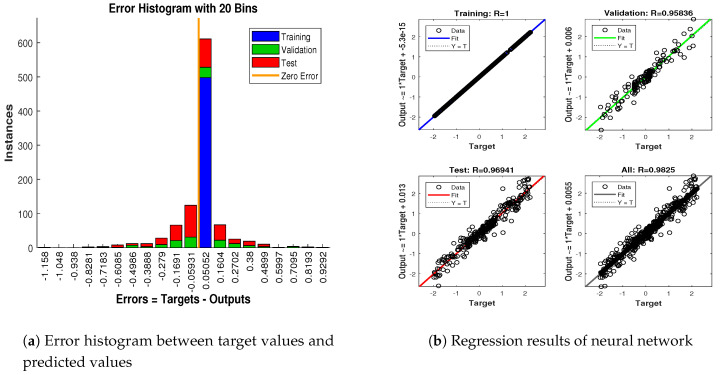
Error histogram and regression of ANN in scenario 6.

In scenario 6, a noisy underwater environment is taken by assuming the highest value of standard deviation of measured noise. Here simulation results are discussed as:

- In Figure 21, state estimates of ANN are analyzed with other two conventional filtering techniques and it is worth to note that ANN is also showing its command even in the cluttered ocean environment.
- In Figure 22, RMSE between true and estimated position of dynamic object is represented which showing that ANN is estimating position of passive dynamic object with less position error.
- In Figure 23a, the error histogram is shown between target time series S(n),S(n−1),...,S(n−j) and estimated value $S_{n}^{Est}$ of target’s position after training the neural network. At the center of the graph, a bin incorporating the error of 0.05052 and the height of that bin for training data set lies near to 600 samples while the validation and test data set lie between 500 and 600 samples. It means that many samples from different data sets have an error that lies in that following range. In this case, the zero error point falls under the bin with center 0.05052.
- In Figure 23b, the regression output of the ANN modeling is presented for training, validation and testing purposes. In the regression results, many divergence points between the actual target and the predicted output appear because this scenario has extremely noisy passive bearings.

Simulation results from all scenarios show that, for higher values of standard deviation of measured noise Ψ, state estimation techniques experience difficulties in approaching the real position of the underwater dynamic object. However, in the comparison between all estimation techniques, neural network intelligence paradigm NARX shows a better performance, which is clearly evident for its effectiveness in nonlinear state estimation problems in an underwater atmosphere.

**Figure 24:**
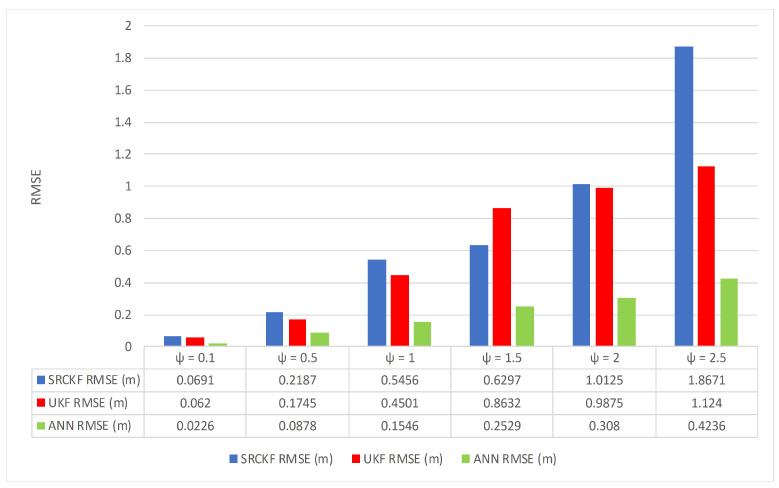
Average RMSEs for estimating the position with SRCKF, UKF and ANN by varying measurement noise.

Along with the simulation results, we also compute RMSE in meters between the actual and predicted position of the target. These position error responses also endorse the above results that the accuracy of ANN is more than double from Kalman filters and it shows the effectiveness of neural network applications in the state estimation of passive target problems. These position errors computed from SRCKF, UKF and ANN are given in Figure 24.

## 5. Conclusions

Intelligent computing through the NARX based neural network is investigated effectively for underwater bearings-only passive state estimation application. The instantaneous position of a passive dynamic target is predicted in two-dimension x-y coordinates at each time instant. Initially, bearings only and state space-based state estimation framework of dynamic and measurement model is mathematically designed. Later, the intelligent computing paradigm based on NARX is designed for the particular problem of the state estimation of the passive object. The NARX based supervised neural network is analyzed for 500 data samples. The competency of neural computing is assessed for semi-curved target movement in the form of minimum root mean square position error. Appropriate numerical values of the white Gaussian measured noise are applied to examine the performance of the proposed methodology. Simulation results clearly depict that the accuracy of the neural network is superior from conventional nonlinear filtering algorithms like SRCKF and UKF. In the noisy underwater scenario, the exponential decay is noticed in the results of all algorithms. Thus, obtaining accurate state estimation in a complex ocean environment is still a challenging task and has wide room for development.

In the future, fractional evolutionary and swarming techniques [50,51,52,53] can be investigated for obtaining better state prediction of highly maneuvering object for non-Gaussian distribution of measurement noise, which is still an exciting research dimension in the state estimation of underwater single or multi targets. This work can also be extended for real implementation of the proposed state estimation scheme.

## Figures and Tables

**Table 1:** State estimation parameters and their suitable values.

Variables	Setting
Initial position and velocity of object	$S_{0}$ = ${\left[-2\;-0.5\;1\;0\right]}^{T}$
Location function of observer elements	$\left(\alpha_{y}^{z},\alpha_{x}^{z}\right)$
Number of sensors	z = 8
Spacing of sensors	0.5
Standard deviation of measurement noise	Ψ=0.1→2.5 radian
Spectral density of process noise	ϖ=0.01
Step size	di=0.01
Number of delays	k = 2
Data points	500
Number of hidden neurons	100
Number of target time step	1000

## References

[1] Ge B., Zhang H., Jiang L., Li Z., Butt M.M. (2019). Adaptive unscented Kalman filter for target tracking with unknown time-varying noise covariance. Sensors.

[2] Li Y., Chen X., Yu J., Yang X. (2019). NA fusion frequency feature extraction method for underwater acoustic signal based on variational mode decomposition, duffing chaotic oscillator and a kind of permutation entropy. Electronics.

[3] Ali W., Li Y., Raja M.A.Z., Ahmed N. (2020). Generalized pseudo Bayesian algorithms for tracking of multiple model underwater maneuvering target. Appl. Acoust..

[4] Patra N., Sadhu S., Ghoshal T.K. (2018). Adaptive state estimation for tracking of civilian aircraft. IET Sci. Meas. Technol..

[5] Fan X., Shang S., Song D., Sun W., Wen Y. (2019). Weak target detection technology of passive radar on the navigation satellite signal. J. Eng..

[6] Hong J., Laflamme S., Dodson J., Joyce B. (2018). Introduction to state estimation of high-rate system dynamics. Sensors.

[7] Wirnshofer F., Schmitt P.S., Meister P., Wichert G.V., Burgard W. State estimation in contact-rich manipulation. Proceedings of the 2019 International Conference on Robotics and Automation (ICRA).

[8] Luo J., Han Y., Fan L. (2018). Underwater Acoustic Target Tracking: A Review. Sensors.

[9] Ahmed H., Ullah I., Khan U., Qureshi M.B., Manzoor S., Muhammad N., Shahid Khan M.U., Nawaz R. (2019). Adaptive Filtering on GPS-Aided MEMS-IMU for Optimal Estimation of Ground Vehicle Trajectory. Sensors.

[10] Li X., Zhao C., Yu J., Wei W. (2019). Underwater Bearing-Only and Bearing-Doppler Target Tracking Based on Square Root Unscented Kalman Filter. Entropy.

[11] Li Y., Cheng Y., Li X., Wang H., Hua X., Qin Y. (2017). Bayesian Nonlinear Filtering via Information Geometric Optimization. Entropy.

[12] Nguyen N.H., Doğançay K. (2017). Multistatic pseudolinear target motion analysis using hybrid measurements. Signal Process..

[13] Pei Y., Biswas S., Fussell D.S., Pingali K. (2019). An elementary introduction to kalman filtering. Commun. ACM.

[14] He Y., Jiang J., Huang H., Zhuo S., Wu Y. (2018). Kalman Filtering Algorithm for Systems with Stochastic Nonlinearity Functions, Finite-Step Correlated Noises, and Missing Measurements. Discret. Dyn. Nat. Soc..

[15] Zhang M., Li K., Hu B., Meng C. (2019). Comparison of Kalman Filters for Inertial Integrated Navigation. Sensors.

[16] Julier S.J., Uhlmann J.K. (1997). New extension of the Kalman filter to nonlinear systems. Signal Process. Sens. Fusion Target Recognit. VI.

[17] Leung H., Zhu Z., Ding Z. (2020). An aperiodic phenomenon of the extended Kalman filter in filtering noisy chaotic signals. Mech. Syst. Signal Process..

[18] Lee Y., Majda A.J. (2016). State estimation and prediction using clustered particle filters. Proc. Natl. Acad. Sci. USA.

[19] Ali W., Li Y., Tanoli S.A.K., Raja M.A.Z., Javaid K., Ahmed N. SConvergence Analysis of Unscented Transform for Underwater Passive Target Tracking in Noisy Environment. Proceedings of the 2019 IEEE International Conference on Signal Processing, Communications and Computing (ICSPCC).

[20] Wang Y., Qiu Z., Qu X. (2017). An improved unscented Kalman filter for discrete nonlinear systems with random parameters. Discret. Dyn. Nat. Soc..

[21] Ali W., Li Y., Chen Z., Raja M.A.Z., Ahmed N., Chen X. (2019). Application of Spherical-Radial Cubature Bayesian Filtering and Smoothing in Bearings Only Passive Target Tracking. Entropy.

[22] Su J., Li Y., Ali W. (2020). Underwater angle-only tracking with propagation delay and time-offset between observers. Signal Process..

[23] Qin W., Wang X., Cui N. (2017). Maximum correntropy sparse Gauss–Hermite quadrature filter and its application in tracking ballistic missile. IET Radar Sonar Navig..

[24] Jia B., Xin M., Cheng Y. (2012). Sparse-grid quadrature nonlinear filtering. Automatica.

[25] Hou J., Yang Y., Gao T. (2019). Variational Bayesian based adaptive shifted Rayleigh filter for bearings-only tracking in clutters. Sensors.

[26] Zhang Z., Fu K., Sun X., Ren W. (2019). Multiple target tracking based on multiple hypotheses tracking and modified ensemble Kalman filter in multi-sensor fusion. Sensors.

[27] Chen X., Li Y., Yu J., Li Y. (2018). Developing the fuzzy c-means clustering algorithm based on maximum entropy for multitarget tracking in a cluttered environment. J. Appl. Remote Sens..

[28] Ahmad I., Zahid H., Ahmad F., Raja M.A.Z., Baleanu D. (2019). Design of computational intelligent procedure for thermal analysis of porous fin model. Chin. J. Phys..

[29] Khan J.A., Raja M.A.Z., Rashidi M.M., Syam M.I., Wazwaz A.M. (2015). Nature-inspired computing approach for solving non-linear singular Emden–Fowler problem arising in electromagnetic theory. Connect. Sci..

[30] Ahmad I., Ahmad S., Awais M., Ahmad S.U.I., Raja M.A.Z. (2018). Neuro-evolutionary computing paradigm for Painlevé equation-II in nonlinear optics. Eur. Phys. J. Plus.

[31] Mehmood A., Zameer A., Ling S.H., Raja M.A.Z. (2018). Design of neuro-computing paradigms for nonlinear nanofluidic systems of MHD Jeffery–Hamel flow. J. Taiwan Inst. Chem. Eng..

[32] Raja M.A.Z., Umar M., Sabir Z., Khan J.A., Baleanu D. (2018). A new stochastic computing paradigm for the dynamics of nonlinear singular heat conduction model of the human head. Eur. Phys. J. Plus.

[33] Sabir Z., Wahab H.A., Umar M., Sakar M.G., Raja M.A.Z. (2020). Novel design of Morlet wavelet neural network for solving second order Lane–Emden equation. Math. Comput. Simul..

[34] Bukhari A.H., Sulaiman M., Islam S., Shoaib M., Kumam P., Raja M.A.Z. (2020). Neuro-fuzzy modeling and prediction of summer precipitation with application to different meteorological stations. Alex. Eng. J..

[35] Raja M.A.Z., Mehmood A., ur Rehman A., Khan A., Zameer A. (2018). Bio-inspired computational heuristics for Sisko fluid flow and heat transfer models. Appl. Soft Comput..

[36] Ahmad I., Raja M.A.Z., Bilal M., Ashraf F. (2017). Neural network methods to solve the Lane–Emden type equations arising in thermodynamic studies of the spherical gas cloud model. Neural Comput. Appl..

[37] Raja M.A.Z., Niazi S.A., Butt S.A. (2017). An intelligent computing technique to analyze the vibrational dynamics of rotating electrical machine. Neurocomputing.

[38] Ahmad I., Ahmad F., Raja M.A.Z., Ilyas H., Anwar N., Azad Z. (2018). Intelligent computing to solve fifth-order boundary value problem arising in induction motor models. Neural Comput. Appl..

[39] Sabir Z., Manzar M.A., Raja M.A.Z., Sheraz M., Wazwaz A.M. (2018). Neuro-heuristics for nonlinear singular Thomas-Fermi systems. Appl. Soft Comput..

[40] Raja M.A.Z., Shah F.H., Tariq M., Ahmad I. (2018). Design of artificial neural network models optimized with sequential quadratic programming to study the dynamics of nonlinear Troesch’s problem arising in plasma physics. Neural Comput. Appl..

[41] Ahmad I., Raja M.A.Z., Bilal M., Ashraf F. (2016). Bio-inspired computational heuristics to study Lane–Emden systems arising in astrophysics model. SpringerPlus.

[42] Esteves J.T., de Souza Rolim G., Ferraudo A.S. (2019). Rainfall prediction methodology with binary multilayer perceptron neural networks. Clim. Dyn..

[43] Hatata A.Y., Eladawy M. (2018). Prediction of the true harmonic current contribution of nonlinear loads using NARX neural network. Alex. Eng. J..

[44] Solanki V., Joshi M. Energy Efficient NARX Model for Target Tracking in Wireless Sensor Network. Proceedings of the 2018 3rd IEEE International Conference on Recent Trends in Electronics, Information & Communication Technology (RTEICT).

[45] Peng J., Tang Q. (2019). Application of NARX Dynamic Neural Network in Quantitative Investment Forecasting System. International Symposium on Intelligence Computation and Applications, Proceedings of the 11th International Symposium, ISICA 2019, Guangzhou, China, 16–17 November 2019.

[46] Pisoni E., Farina M., Carnevale C., Piroddi L. (2009). PForecasting peak air pollution levels using NARX models. Eng. Appl. Artif. Intell..

[47] Raptodimos Y., Lazakis I. (2020). Application of NARX neural network for predicting marine engine performance parameters. Ships Offshore Struct..

[48] Liu J., Li T., Zhang Z., Chen J. (2020). NARX prediction-based parameters online tuning method of intelligent PID system. IEEE Access.

[49] Rangel E., Cadenas E., Campos-Amezcua R., Tena J.L. (2020). Enhanced Prediction of Solar Radiation Using NARX Models with Corrected Input Vectors. Energies.

[50] Zameer A., Muneeb M., Mirza S.M., Raja M.A.Z. (2020). Fractional-order particle swarm based multi-objective PWR core loading pattern optimization. Ann. Nucl. Energy.

[51] Muhammad Y., Khan R., Ullah F., ur Rehman A., Aslam M.S., Raja M.A.Z. (2019). Design of fractional swarming strategy for solution of optimal reactive power dispatch. Neural Comput. Appl..

[52] Akbar S., Zaman F., Asif M., Rehman A.U., Raja M.A.Z. (2019). Novel application of FO-DPSO for 2-D parameter estimation of electromagnetic plane waves. Neural Comput. Appl..

[53] Lodhi S., Manzar M.A., Raja M.A.Z. (2019). Fractional neural network models for nonlinear Riccati systems. Neural Comput. Appl..

